# Zinc finger nuclease‐mediated targeting of multiple transgenes to an endogenous soybean genomic locus via non‐homologous end joining

**DOI:** 10.1111/pbi.13012

**Published:** 2018-10-15

**Authors:** Nicholas D. Bonawitz, W. Michael Ainley, Asuka Itaya, Sivarama R. Chennareddy, Tobias Cicak, Katherine Effinger, Ke Jiang, Tejinder Kumar Mall, Pradeep Reddy Marri, J. Pon Samuel, Nagesh Sardesai, Matthew Simpson, Otto Folkerts, Rodrigo Sarria, Steven R. Webb, Delkin O. Gonzalez, Daina H. Simmonds, Dayakar R. Pareddy

**Affiliations:** ^1^ Dow AgroSciences LLC Indianapolis IN USA; ^2^ Agriculture and Agri‐Food Canada Ottawa ON Canada; ^3^ Present address: Genus IntelliGen Technologies Windsor WI USA

**Keywords:** genome editing, gene targeting, zinc finger nuclease, soybean, somatic embryogenesis, biolistic transformation

## Abstract

Emerging genome editing technologies hold great promise for the improvement of agricultural crops. Several related genome editing methods currently in development utilize engineered, sequence‐specific endonucleases to generate DNA double strand breaks (DSBs) at user‐specified genomic loci. These DSBs subsequently result in small insertions/deletions (indels), base substitutions or incorporation of exogenous donor sequences at the target site, depending on the application. Targeted mutagenesis in soybean (*Glycine max*) via non‐homologous end joining (NHEJ)‐mediated repair of such DSBs has been previously demonstrated with multiple nucleases, as has homology‐directed repair (HDR)‐mediated integration of a single transgene into target endogenous soybean loci using CRISPR/Cas9. Here we report targeted integration of multiple transgenes into a single soybean locus using a zinc finger nuclease (ZFN). First, we demonstrate targeted integration of biolistically delivered DNA via either HDR or NHEJ to the *FATTY ACID DESATURASE 2‐1a* (*FAD2‐1a*) locus of embryogenic cells in tissue culture. We then describe ZFN‐ and NHEJ‐mediated, targeted integration of two different multigene donors to the *FAD2‐1a* locus of immature embryos. The largest donor delivered was 16.2 kb, carried four transgenes, and was successfully transmitted to T_1_ progeny of mature targeted plants obtained via somatic embryogenesis. The insertions in most plants with a targeted, 7.1 kb, NHEJ‐integrated donor were perfect or near‐perfect, demonstrating that NHEJ is a viable alternative to HDR for gene targeting in soybean. Taken together, these results show that ZFNs can be used to generate fertile transgenic soybean plants with NHEJ‐mediated targeted insertions of multigene donors at an endogenous genomic locus.

## Introduction

Genome editing is the introduction of heritable genetic changes at user‐specified locations in the genome. Types of genetic changes that can be induced include gene disruption, gene excision, sequence substitution and targeted integration of exogenous sequence (Carroll, 2014; Kim and Kim, 2014; Sander and Joung, 2014; Weeks *et al*., 2016). The ability to edit or insert genes at target loci is valuable for a variety of applications such as elucidation of gene function, treatment of genetic diseases, and genetic improvement of plants, animals or microbes for industry or agriculture.

Genetically engineered crops provide farmers with a suite of options for controlling or mitigating the effects of weeds, pest insects, diseases and abiotic stresses. The development of new commercial transgenic traits is, however, cost‐ and time‐intensive, as it involves (i) the generation and phenotypic screening of many independent transgenic events, each with different random transgene insertions, (ii) introgression of transgenic loci from selected events to elite germplasm and (iii) repeated monitoring of transgenes for maintained expression and efficacy. Genome editing technologies promise to improve this paradigm by enabling targeted transgene integration into genomic loci known to support robust and stable gene expression and known not to interfere with agronomic characteristics, thus facilitating the identification of events that perform as desired (Davies *et al*., 2017; Petolino and Kumar, 2016; Sun *et al*., 2016). Targeting multiple transgenes into a single characterized locus also simplifies their introgression into elite germplasm, a task that becomes exponentially more complex as the number of independently segregating loci increases.

Central to most recent genome editing efforts is the creation of one or more DNA DSBs by an engineered endonuclease (also referred to as site‐directed nucleases or sequence‐specific nucleases). Engineered nucleases currently in use include meganucleases, TALENs (Tal effector‐like endonucleases), ZFNs and a collection of related CRISPR/Cas (clustered, regularly interspaced palindromic repeats/CRISPR‐associated) ribonucleoproteins (Carroll, 2014; Kim and Kim, 2014; Weeks *et al*., 2016). All of these engineered nucleases have in common that they couple a sequence‐independent endonuclease activity with a programmable DNA binding activity that is sufficiently specific to target a unique genomic site. In the case of ZFNs, DNA binding is provided by an array of four to six synthetic zinc finger domains with a corresponding 12–18 bp binding site, and nuclease activity is provided by the endonuclease domain of *Fok*I (Urnov *et al*., 2010). Due to the dimerization‐dependent activity of *Fok*I (Bitinaite *et al*., 1998), ZFNs are used in pairs, with one member of each pair binding on either side of a target site. The use of two obligately heterodimerizing “high‐fidelity” (or “HiFi”) *Fok*I domains on ZFN monomers of a particular pair prevents monomer self‐association and reduces off‐target endonuclease activity (Doyon *et al*., 2011; Miller *et al*., 2007; Szczepek *et al*., 2007).

Engineered nucleases have been successfully used to edit the genomes of numerous plant species by stimulating either NHEJ‐ or HDR‐mediated repair at the site of the induced DSB (Songstad *et al*., 2017; Pacher and Puchta 2017; Weeks *et al*., 2016; Steinert *et al*., 2016; Sun *et al*., 2016; Voytas 2013). NHEJ involves the direct ligation of two DNA ends to one another and is thought to be the primary mechanism of DSB repair in most plant tissues (Puchta, 2005). In contrast, HDR is prevalent only in actively dividing cells, and involves replication‐mediated copying of sequence from any genomic or exogenously provided DNA that possesses sequences homologous to those flanking the DSB (Boyko *et al*., 2006; Hiom, 2010). As in genome editing efforts in animals, NHEJ has primarily been used in plants to disrupt or excise target genes, whereas most attempts at targeted gene replacement, modification or integration have utilized donors designed for HDR, with sequences to be delivered to the genome flanked by homology arms of a few hundred to a few thousand base pairs (Kim and Kim, 2014; Maruyama *et al*., 2015; Sander and Joung, 2014). HDR‐delivered sequences have in most cases been relatively short, consisting of either a single transgene or a smaller DNA element. Exogenous DNA has been successfully integrated via HDR into both endogenous loci (D'Halluin *et al*., 2013; Li *et al*., 2015; Shukla *et al*., 2009; Svitashev *et al*., 2015) and into pre‐integrated transgenic target sites (Ainley *et al*., 2013; Schneider *et al*., 2016), with the latter approach representing the proof‐of‐concept for sequential trait stacking strategies. The methods used to deliver donor DNA and nucleases to target cells, to identify and/or select for targeted cells, and to regenerate mature plants from targeted cells have varied widely.

Soybean (*Glycine max*) is a globally important oilseed crop, most current commercial varieties of which are transgenic (ISAAA, 2016). While targeted gene disruption has been demonstrated in this species using various nucleases (Curtin *et al*., 2011; Du *et al*., 2016; Haun *et al*., 2014; Jacobs *et al*., 2015; Sun *et al*., 2016), reports of targeted transgene integration have been limited. The earliest such report described the use of Flippase (FLP) recombinase to deliver a two‐gene donor to a pre‐integrated Flippase recognition target (*FRT*) site, with donors designed to select for targeted integration (Li *et al*., 2009). More recently, (Li *et al*., 2015) demonstrated HDR‐mediated, targeted integration of a single transgene to a CRISPR/Cas‐induced DSB at an endogenous locus in the absence of selection for targeted insertion. We previously reported a set of ZFNs capable of generating DSBs at the endogenous soybean *FAD2‐1a* locus (Glyma.10g278000; Ainley *et al*., 2016), which encodes a fatty acid desaturase involved in determining seed oil profile (Pham *et al*., 2010). Here we report the use of one of these ZFNs to deliver several different DNA donors to this locus. The largest donor delivered carried four transgenes, lacked significant homology to the *FAD2‐1a* target site, and was faithfully transmitted to progeny of primary transformants. These data show that ZFNs can be used to deliver DNA donors to target soybean loci, extend the size of donors that can be targeted, and demonstrate that donor integration can be mediated by NHEJ, potentially expanding the range of tissues available for transgene targeting.

## Results

### ZFN‐mediated, targeted integration of a single‐transgene donor to the *FAD2‐1a* locus of embryogenic tissue culture cells via HDR

**Figure 1:**
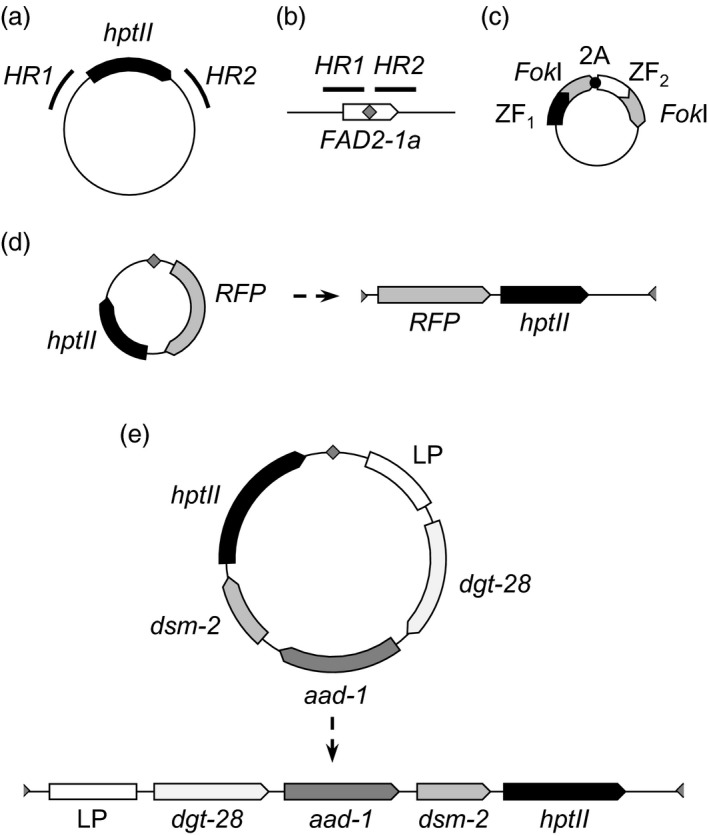
Schematic of targeting constructs and target site. (a) HDR donor. *hptII* denotes the hygromycin phosphotransferase gene and its regulatory elements; *HR1* and *HR2* are 1 kb homology arms. (b) *FAD2‐1a* locus, showing the relative position of the *FAD2‐1a* coding sequence (white arrow), ZFN target site (grey diamond) and homology arms (*HR1* and *HR2*). (c) ZFN expression construct, showing the configuration of zinc finger arrays (ZF1 and ZF2), *Fok*I domains and 2A ribosomal stutter sequence. (d) 7.1 kb NHEJ donor before and after linearization at the ZFN target site. RFP: red fluorescent protein gene. (e) 16.2 kb, four‐gene donor, before and after linearization at the ZFN target site. LP, landing pad; dgt‐28, glyphosate tolerance marker; aad‐1, 2,4‐D tolerance marker; dsm‐2, glufosinate tolerance marker. The thin, black lines and half‐diamonds flanking the transgenes in (d) and (e) represent vector backbone and ZFN monomer‐binding sites respectively.

To quickly and cost‐effectively test various experimental designs for ZFN‐mediated transgene integration, we conducted preliminary experiments in soybean embryogenic cultures. These cultures are amenable to biolistic transformation, antibiotic selection, clonal propagation, and in some cases, regeneration of mature plants (Simmonds, 2003). Due to the widespread use of HDR for gene targeting, as well as the previous report of HDR‐mediated targeting using CRISPR‐Cas (Li *et al*., 2015), we initially attempted to deliver a single‐transgene donor via HDR. This donor consisted of a single selectable marker gene (hygromycin phosphotransferase; *HPT*; Fig. 1a) driven by a constitutive promoter and flanked on either side by ~1 kb of genomic sequence from the region flanking the ZFN target site (Fig. 1b). The design of the ZFN expression constructs (Fig. 1c) was similar to others we have described previously (Ainley *et al*., 2016; Shukla *et al*., 2009), with both ZFN monomer polypeptides generated from a single constitutively expressed transcript by virtue of the T2A ribosomal stutter sequence (Miller *et al*., 2007; Szymczak *et al*., 2004).

To generate cells which both expressed the FAD2‐specific ZFN and which contained the *FAD2‐1a* HDR donor, we co‐bombarded embryogenic tissue cultures with both the HDR donor and ZFN expression constructs. The resulting hygromycin‐resistant clones were then screened for the presence of *HPT* via qPCR, with those verified to contain *HPT* being selected for further analysis. As our experimental design included no specific selection for targeted integration at *FAD2‐1a*, the majority of *HPT* integration events were anticipated to have occurred at non‐target loci in the genome. To identify candidate events containing a targeted integration of *HPT* at *FAD2‐1a*, we analysed genomic DNA from each clone using an endpoint PCR assay for the 5′ and 3′ novel genome‐donor junctions that yields an amplicon of an expected size only if the donor has integrated at the target locus.

Our initial junction PCR analyses were deliberately permissive, with minimization of false negatives given priority over minimization of false positives (since false positives could be subsequently identified with more stringent analyses, whereas false negatives would have been discarded in our experimental design). Specifically, primers were designed to bind just outside the identical homology regions present in both the target and the donor. The use of these primers thus yields the shortest and most easily amplified products capable of detecting targeted transgene integrations, but will detect both full‐length and partial transgene integrations with internal deletions. Furthermore, although the sequence of each primer binding site was unique in the soybean genome, primers were not designed specifically to maximize differences with the corresponding sites in paralogous loci, and thus some apparent positives may be from targeted integrations at a locus paralogous to *FAD2‐1a*.

As shown in Table 1 (experiments 1 and 2), from a total of 1653 clones analysed from two independent experiments testing multiple experimental parameters, we identified 47 clones that gave rise to at least one junction amplicon indicating targeted integration of donor DNA. None of the experimental parameters we tested (e.g. particle size or homodimerizing vs heterodimerizing ZFNs) exhibited a statistically significant effect on the frequency with which hygromycin‐resistant clones yielded such junction amplicons, as the calculated 90% binomial confidence interval associated with this frequency overlapped for all experimental treatments. On the basis of these observations, we tentatively concluded that ZFN‐ and HDR‐mediated, targeted integration of at least a partial DNA donor had occurred in a few per cent of clones analysed, across multiple different experimental treatments.

**Table 1:** ZFN‐mediated, targeted transgene integration in soybean tissue culture

Exp.	Gold dia. (μm)	ZFN	Donor	ZFN:donor	Cultivar	Hyg^R^ clones analysed	Clones w/junction amplicon	Targeting efficiency (%)
1–1	1.0	Homo	HDR	1:5	X5	99	2	0.4––6.2
1–2	1.0	Hetero	HDR	2:5	X5	67	0	0.0––4.4
1–3	0.6	Homo	HDR	1:5	X5	159	8	2.5––8.9
1–4	0.6	Hetero	HDR	2:5	X5	365	7	0.9––3.6
2–1	0.6	Homo	HDR	1:5	X5	521	10	1.0––3.2
2–2	0.6	Homo	HDR	2:5	X5	442	20	3.0––6.5
3–1	0.6	Homo	NHEJ	1:5	Westag 97	87	16	11.9––26.6
3–2	0.6	Homo	NHEJ	1:5	X5	228	42	14.3––23.2
3–3	0.6	Homo	NHEJ	2:5	Westag 97	120	15	7.9––18.6
3–4	0.6	Homo	NHEJ	2:5	X5	328	60	14.9––22.2

Exp., Experiment number; gold dia., diameter of gold particles used for bombardment; ZFN, *Fok*I configuration of ZFN monomers (homo = homodimerizing, hetero = heterodimerizing); ZFN:donor, molar ratio of ZFN expression construct to donor construct; cultivar, soybean cultivar source of suspension cells; hyg^R^, hygromycin‐resistant; targeting efficiency, 90% confidence interval of targeting efficiency.

### ZFN‐mediated, targeted integration of a multigene donor to the *FAD2‐1a* locus of embryogenic tissue culture cells via NHEJ

#### Extensive homology of the donor to the target locus is dispensable for ZFN‐mediated targeted transgene integration

Having obtained preliminary evidence for HDR‐mediated targeting, we next tested whether the regions of sequence identity flanking the desired donor region (i.e. the homology arms) are required for targeted transgene integration. Specifically, we aimed to determine whether donor DNA could be delivered to a target locus via NHEJ, as this DNA repair mechanism (but not HDR) is thought to be operable in most cells at most times (Puchta, 2005), and thus might represent a means by which to increase the range of cell or tissue types available for targeting.

For these experiments, we made several modifications to the DNA donor to facilitate integration via NHEJ (Fig. 1d). In contrast with HDR‐mediated gene targeting, in which a circular donor can serve as a template for synthesis of new DNA at the target site, NHEJ‐mediated gene targeting involves direct incorporation of the donor, which must first be linearized via the formation of at least one DSB. We therefore incorporated into the circular NHEJ donor construct a single target site for the same ZFN pair used to generate the DSB at *FAD2‐1a*. In addition, we incorporated a second transgene (encoding a red fluorescent protein; RFP) into the NHEJ donor construct (Fig. 1d) to simultaneously test our ability to deliver a larger, 7.1 kb DNA fragment to the target locus.

Embryogenic tissue cultures were transformed and screened for *HPT* as in the HDR‐based experiments above, then screened by endpoint PCR for the presence of novel genome‐donor junctions, taking into account that the NHEJ donor could be incorporated in either of the two possible orientations. From 763 hygromycin‐resistant clones analysed, 133 yielded at least one junction amplicon under the permissive PCR conditions, providing strong preliminary evidence that extended homology regions flanking the donor are not necessary for targeted integration (Table 1, experiment 3). We again found that none of the experimental variables we evaluated (e.g. molar ratio of donor to ZFN expression vector or cultivar source of tissue cultures) had a statistically significant effect on the frequency with which hygromycin‐resistant clones yielded junction amplicons. On the other hand, the overall frequency with which we obtained junction amplicons when using the NHEJ donor was apparently higher than the corresponding frequency for the HDR donor, with the 90% binomial confidence intervals for this frequency overlapping for only a single NHEJ‐ and a single HDR‐based experimental treatment. Although the NHEJ‐ and HDR‐based experiments were not performed in parallel, a straightforward interpretation of the data is that the efficiency of NHEJ‐based targeting in this experimental system is as high as or higher than HDR‐based targeting.

#### Overlapping PCR and sequence analysis confirm multiple events with full‐length, single copy, targeted transgene integrations

From the amplicon‐positive, candidate targeted events described in the previous two sections, we selected 95 low or single copy events for additional molecular analysis (62 from NHEJ‐based targeting and 33 from HDR‐based targeting). First, genomic DNA from each clone was analysed by junction PCR using a second set of genome‐ and donor‐specific primers that had been designed for higher stringency. Specifically, genomic primers were designed and verified to generate amplicons from *FAD2‐1a* but not from the most closely related paralog *FAD2‐1b*. The binding sites of these primers were more distant from the ZFN target site (>1.5 kb) such that they would detect small‐scale deletions or rearrangements near the site of the ZFN‐induced DSB. In addition, donor primers were designed and verified to bind to an internal region of the *HPT* gene, such that the entire sequence of an intact targeted donor (either HDR or NHEJ) would be contained within an overlapping pair of genome‐donor junction amplicons.

**Figure 2:**
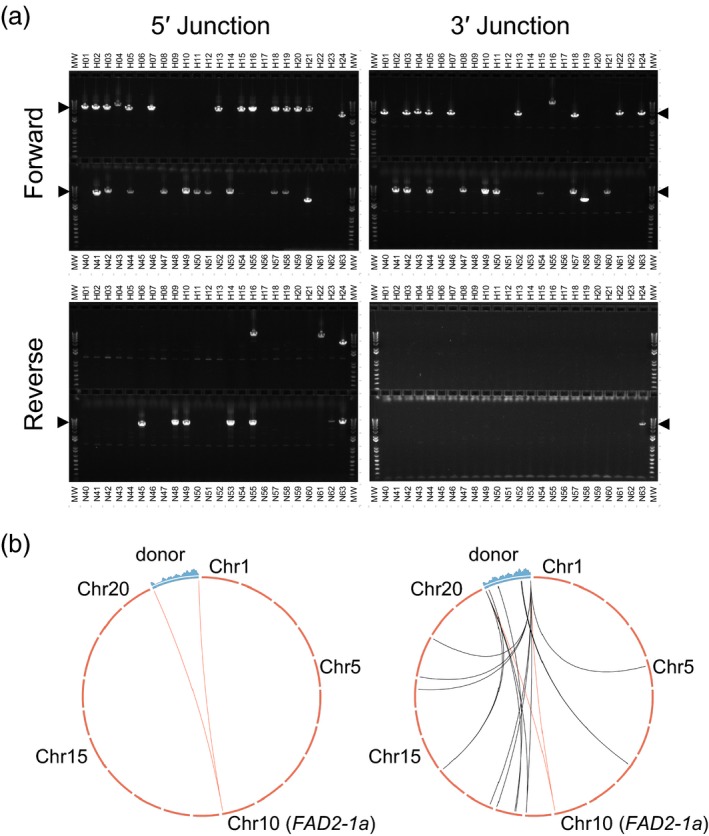
HDR‐ and NHEJ‐mediated targeting in embryogenic tissue culture. (a) Representative stringent junction PCR results on 24 candidate events each from HDR‐based targeting experiments (top row of each gel) or NHEJ‐based targeting experiments (bottom row of each gel). 5′, 3′, forward and reverse are all with respect to the *FAD2‐1a* target locus. Black arrowheads indicate the expected size of an amplicon from a fully intact donor. HDR‐based targeting is not anticipated to give rise to donor integration in reverse orientation. (b) Circos plots (http://circos.ca) showing NGS read coverage across the donor (blue histogram above the corresponding section of the genetic map) and deduced donor integration sites (arcs) for a single copy (left) and a complex multicopy transgenic event (right). The red arc indicates the desired insertion site at *FAD2‐1a*; the black arcs indicate off‐target insertions.

From the 95 clones analysed under these more stringent conditions, a total of 56 (37 NHEJ, 19 HDR) gave rise to an amplicon of the expected size for at least one genome‐donor junction (Fig 2a). The orientation of integration in the great majority of events was such that the transcription of *HPT* (and *RFP*, if present) was in the same direction as the transcription of *FAD2‐1a* (denoted “forward” orientation). This orientation is also such that in the case of perfect NHEJ‐mediated integration, two functional ZFN target sites are reconstituted flanking the donor. Interestingly, several integration events for the HDR donor were apparently in reverse orientation, suggesting that despite the presence of homology arms, these donors were integrated at the target locus via NHEJ.

Of the 56 events giving rise to at least one amplicon of expected size, 29 (19 NHEJ, 10 HDR), gave rise to a single 5′ and a single 3′ amplicon of expected size for a full‐length, targeted integration. The remainder variously yielded no amplicons, a single amplicon, amplicons of unexpected size, or two or more amplicons associated with conflicting donor orientations, indicating that donors were fragmented, concatemerized, or otherwise aberrant, or that the initial PCR was a false positive.

In tandem with stringent junction PCR, all 95 clones were also analysed by Illumina‐based Next Generation Sequencing. Prior to paired‐end sequencing, DNA elements of interest (HDR donor, and NHEJ donor) and adjacent sequences were enriched using sequence capture—isolation of selected DNA fragments via hybridization to bead‐bound oligonucleotides complementary to these sequences. To identify events in which targeted integration had occurred in the NHEJ‐based experiment, we searched the set of all reads from each sample for individual reads that spanned the novel genome‐donor junction created by targeted integration (Fig. 2b). Of the 62 events from the NHEJ‐based experiment thus analysed, 42 were found to contain at least one junction indicative of targeted integration (either of full‐length or partial donor), 21 of which contained two junctions consistent with integration of a full‐length donor at *FAD2‐1a*. Donor intactness could be directly demonstrated in the four of these 21 events containing only a single copy of the donor, as reads covering the entire donor can be identified and uniquely attributed to the single integration at *FAD2‐1a*. For the remaining 17 events, the presence of multiple donors (either on‐ or off‐target) prevents the assignment of internal donor reads to any particular copy of the donor, and thus prevents donor intactness from being directly assessed via NGS in these events.

The results of NGS and high stringency PCR of the NHEJ events were in close agreement with one another (Table S1). Of the 63 junctions identified by NGS and indicating targeted insertions as described above, 51 were directly supported by the detection of a corresponding full‐length junction amplicon in high stringency PCR. The other 12 junctions not PCR amplified may not have had intact primer binding sites or the PCR reaction may have failed for other reasons. NGS failed to identify nine junctions expected from events with apparent full‐length junction amplicons.

NGS analysis of HDR‐derived clones was used to determine the existence of off‐target insertions, as well as to address donor intactness in the case of clones where all genome‐donor junctions mapped to a single genomic locus. These analyses showed that five of the ten clones exhibiting single 5′ and 3′ junction amplicons of expected size in stringent junction PCR had no detectable off‐target integrations, and no evidence for fragmentation of the donor at *FAD2‐1a*. The donor integrations in these five events can thus be inferred to be intact, and the lack of detectable integration at other sites in the genome can be taken as indirect evidence for integration at *FAD2‐1a*. It should be noted, however, that because the region of shared sequence in the donor and the target site (1000 bp) was longer than the fragments sequenced by NGS (~800 bp) no individual reads contained sequence that was unambiguously attributable to both the genome and the donor.

### ZFN‐mediated, targeted integration of multigene donors at the FAD2‐1a locus of soybean embryos via NHEJ, regeneration of mature plants, and transmission of donors to progeny

#### ZFN‐mediated, targeted integration of a 7.1 kb, two‐transgene donor in immature embryo‐derived somatic embryos via NHEJ

**Figure 3:**
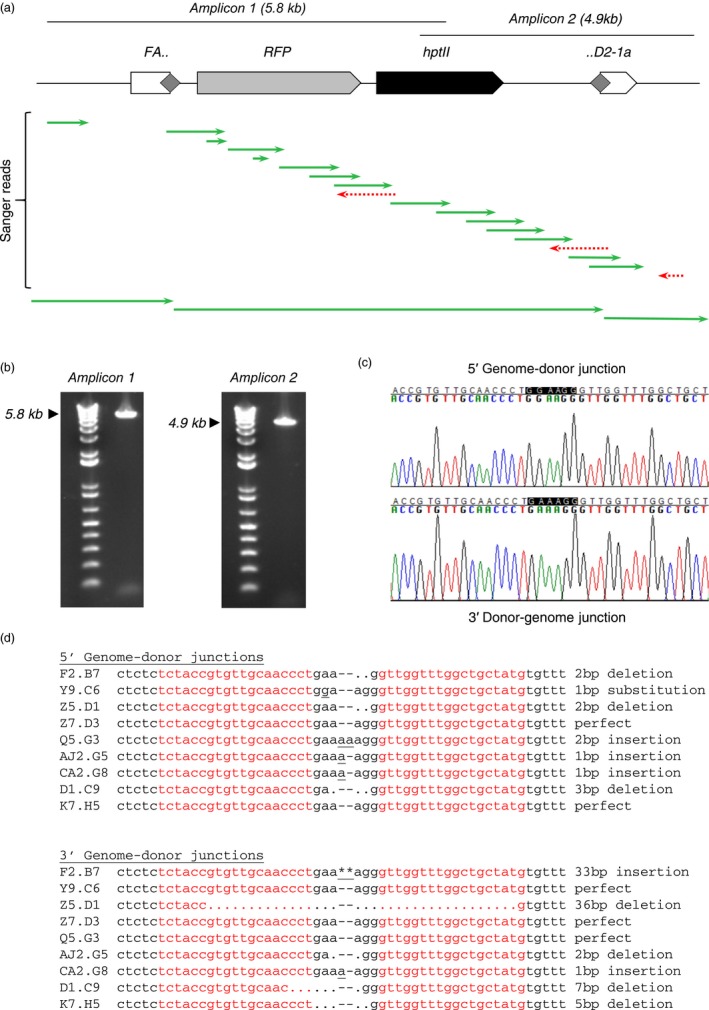
NHEJ‐mediated targeting in somatic embryogenesis‐derived mature plants. (a) Position of overlapping, genome‐anchored junction amplicons in relation to a fully intact, 7.1 kb donor targeted to *FAD2‐1a*, with overlapping Sanger sequencing reads from a representative targeted event (event F2.B7). Grey diamonds represent the ZFN target site, duplicated in the case of a perfectly integrated donor. (b) Visualized DNA amplicons from the same representative sample shown in (a). Expected product sizes are marked with a black arrowhead. (c) Sanger sequencing‐derived chromatograms corresponding to the genome‐donor junctions of a representative targeted event (event Y9.C6). The sequence of the perfect junction is GAAAGG. This event has a single nucleotide substitution (A to G) at the 5′ genome‐donor junction and a perfect 3′ donor‐genome junction. (d) Sequences of both genome‐donor junctions from all nine fully intact targeted events obtained. The sequences of the individual ZFN monomer‐binding sites are shown in red.

Having concluded that ZFNs can drive NHEJ‐mediated targeted transgene integration in tissue cultures, we next tested whether we could reproduce these results using established methods for generating transgenic soybean plants. Specifically, we attempted to generate, via biolistic bombardment of split immature embryos and subsequent somatic embryogenesis, stable transgenic plants with the full‐length, 7.1 kb HPT‐ and RFP‐expressing NHEJ donor precisely integrated at the *FAD2‐1a* locus (Fig. 3a).

Immature embryos excised from developing soybean pods were transformed with the NHEJ donor construct and either the homodimerizing ZFN or heterodimerizing ZFN expression construct. These embyros were then cultured on a series of media to induce somatic embryogenesis, select for transformants, and induce root and shoot formation, after which they were transferred to soil. Leaf samples for DNA extraction and molecular analysis were harvested from regenerated shoots non‐destructively after they had accumulated enough biomass to do so with minimal risk of killing the developing plantlet.

Regenerated shoots were screened for the presence of the *HPT* gene, and *HPT*‐positive events screened by junction PCR as above. From a total of 5802 regenerated shoots, 326 were positive for *HPT* and were therefore true transformants (Table 2). This apparently high number of escapes was anticipated, and is consistent with our previous observations that relatively low‐stringency hygromycin selection increases the frequency with which somatic embryos can be successfully regenerated into mature plants at the cost of a relatively high escape rate. Of the 326 *HPT*‐positive shoots, 32 yielded junction amplicons (Table 2). A number of these events showed evidence of donor fragmentation or artefactual amplification from tandem insertions. However, in addition to these non‐desired plants, we also identified a total of 12 that had a single 5′ and a single 3′ amplicon consistent with full‐length, targeted insertions (Fig. 3b,c), nine of which were verified by Sanger sequencing to be fully intact. The majority of these nine plants had indels of less than 10 bp at both genome‐donor junctions, with only two longer indels found among the 18 sequenced genome‐donor junctions (a single 33 bp insertion and a single 36 bp insertion). Five of the 18 sequenced junctions were perfect, reconstituting a ZFN cut site identical to that in both the genomic target site and the NHEJ donor construct (Fig 3d).

**Table 2:** ZFN‐mediated, targeted transgene integration in regenerated plants

ZFN	Donor size (kb)	IE bombarded	IE forming shoots	Total regenerated shoots	*HPT+* shoots	Shoots w/junction amplicon	Shoots w/intact donor at target	Mature plants w/intact donor at target
Homo	7.1	5050	933	2708	77	6	2	2
Hetero	7.1	5100	961	3094	249	26	7	3
Hetero	16.2	10230	2948	9824	1290	205	3	3

ZFN, *Fok*I configuration of ZFN monomers (homo = homodimerizing, hetero = heterodimerizing); IE, immature embryos.

#### Generation of mature plants carrying a targeted 16.2 kb, four‐gene donor, and transmission to T_1_ progeny

**Figure 4:**
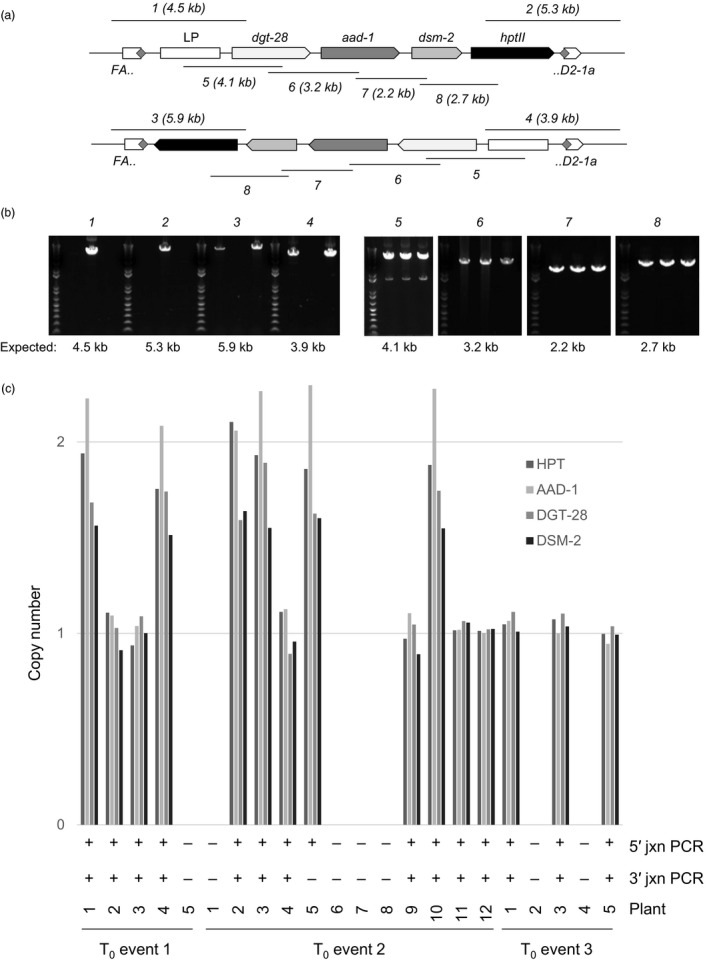
NHEJ‐mediated targeting and Mendelian transmission of a 16.2 kb, four‐gene donor. (a) Position of genome‐anchored junction amplicons (*1‐4*) and internal amplicons (*5‐8*) in relation to a fully intact 16.2 kb donor targeted to *FAD2‐1a* in either forward orientation (top) or reverse orientation (bottom). (b) Detection of junction amplicons and internal amplicons in three independent, fully intact, targeted T_0_ events. The three events are shown in the same order in each gel, but not individually labelled for clarity. Detection of amplicons *1* and *2* in event 2 indicates that the donor has integrated in the forward orientation, whereas detection of amplicons *3* and *4* in events 1 and 3 indicates that the donor has integrated in reverse orientation. All three events yielded all four internal donor amplicons. (c) Targeted insertions were faithfully transmitted to T_1_ progeny. Summarized are the results of junction PCR and qPCR for all four donor‐associated genes for 22 T_1_ progeny of the three T_0_ events shown in (b). T_0_ events 1 and 3 produced a total of five viable T_1_ progeny each, the analytical results of which are shown. The co‐segregation of all four qPCR signals and both junction amplicons is nearly perfect.

To build on the results we obtained with the 7.1 kb, two‐transgene donor described above, and to attempt to deliver a donor of a size more typical for commercial molecular transgene stacks currently being developed (Petolino and Kumar, 2016), we designed a new donor carrying four transgenes—HPT plus three genes conferring herbicide tolerance, as well as a synthetic “landing pad” containing previously validated ZFN recognition sites for further rounds of targeting (Ainley *et al*., 2013). This donor was 16.2 kb in length, and was similar to the 7.1 kb donor in design, containing a single recognition site for the *FAD2‐1a*‐specific ZFN (Figs 1e and 4a). Co‐bombardment of immature embryos with the 16.2 kb donor and the *FAD2‐1a*‐specific ZFN expression construct, somatic embryogenesis, selection for hygromycin resistance, regeneration of plantlets and qPCR screening for HPT were all carried out identically to the experiments with the 7.1 kb donor described above. The results of these experiments are reported in Table 2. From a total of 10 230 immature embryos bombarded, 2948 gave rise to 9824 regenerated shoots (i.e. some immature embryos yielded more than one somatic embryo). From these, 1290 were found by qPCR to contain HPT, and were further screened by endpoint junction PCR. For these junction PCR reactions, we used the same *FAD2‐1a* genomic primers as above, and one of the same donor‐specific HPT primers, but given the large size of the donor, designed an additional donor‐specific primer to screen for the junction distal to HPT. From the 1290 events screened, 205 gave rise to at least one junction amplicon. Consistent with our previous observations that fragmentation can occur with large donors, many events gave rise to either a single 5′ or 3′ amplicon, or to amplicons of sizes inconsistent with integration of a full‐length, intact donor. Nevertheless, we identified three events that gave rise to a single 5′ and a single 3′ amplicon of sizes consistent with full‐length targeted integration (Fig. 4b). One of the events had integrated the donor in forward orientation (i.e. the transcription of the donor‐encoded transgenes was in the same direction as *FAD2‐1a*), and the remaining two events had integrated the donor in reverse orientation.

Each of the three candidate targeted plantlets was transferred to soil and allowed to grow to maturity. Junction PCR reactions on DNA resampled from newly developing tissue confirmed the presence of the expected 5′ and 3′ amplicons, and qPCR‐based copy number analysis of the same DNA showed that each of the four transgenes of the donor construct was present as a single copy in each of the three developing plants, consistent with a single, fully intact targeted insertion in each. The same primers used for qPCR were used to generate five overlapping PCR amplicons covering the entire length of the donor. The sizes of these amplicons, as well as their sequences (as determined by Sanger sequencing) confirmed the presence of a full‐length targeted insertion in each of the three events (Fig. 4b). Finally, each of these three plants was allowed to reproduce via self‐fertilization, the resulting seeds germinated, and the newly emerged seedlings analysed by junction PCR and qPCR. For all three events, both the donor‐encoded transgenes and the junction amplicons were detected in the T_1_ progeny, and displayed perfect co‐segregation of the transgenes with one another and with both junction amplicons, with the exception of a single plant (Fig. 4c). From these data, we conclude that these three plants and their progeny represent successful ZFN‐ and NHEJ‐mediated targeting of a four gene, 16.2 kb donor to an endogenous soybean locus.

## Discussion

We have reported here first use of an engineered, site‐specific endonuclease to deliver large, multigene donors to a target genomic locus of a major crop via NHEJ. Specifically, we have shown that biolistic bombardment of soybean cells with a ZFN expression construct targeting a site in *FAD2‐1a*, together with a circular DNA donor bearing the same target site, is sufficient to drive precise integration of the donor into the target locus. Targeted integration was achieved in both tissue culture and in immature embryos, followed in the latter by somatic embryogenesis, regeneration of fertile plants and Mendelian transmission of the targeted insertion from primary transformants to their progeny. The largest donor integrated was approximately 16.2 kb, carried four transgenes, lacked any shared sequence with the genome besides the ZFN target site and was integrated in either of the two possible orientations relative to the target. For all configurations of donor and ZFN expression construct tested, bombardment of 5 000 to 10 000 immature embryos was sufficient to yield several mature plants with fully intact targeted integrations of the donor. These results show that homology arms are dispensable for targeted transgene integration in plants, that donors sufficiently large to accommodate multiple transgenes can be delivered to an endogenous locus of a crop plant using ZFNs and that the frequency with which mature targeted soybean plants can be generated with this system is compatible with commercial production.

The 7.1 and 16.2 kb multigene donors we have reported here lacked the ~1 kb homology arms that are typically used in HDR‐based gene targeting experiments in both plants and animals. The decision to exclude these homology arms from constructs delivered to immature embryos was based on our initial experiments with tissue cultures, in which events with putative junction amplicons were recovered at a higher frequency with donors lacking homology arms than with donors possessing homology arms (Table 1). Experiments in immature embryos confirmed that the homology arms are dispensable for targeted transgene integration in this system. Together with the pattern of indels we observed at genome‐donor junctions and the fact that donors were integrated into the genome in either possible orientation, we conclude that donors lacking homology arms were incorporated into the genome via NHEJ. Consistent with this conclusion, NHEJ‐mediated targeting with ZFNs has previously been reported in both mammalian tissue culture cells (Orlando *et al*., 2010) and in Arabidopsis (Weinthal *et al*., 2013), though in the latter at a frequency far lower than that reported here. Whether the increased frequency with which we recovered targeted plants relative to Weinthal *et al*. is attributable to species, transformation system, nuclease activity or another variable remains an open question.

In systems where it functions efficiently, NHEJ‐mediated targeting offers several advantages over HDR‐mediated targeting. First, because NHEJ is the primary mechanism of DSB repair in plant somatic cells (Puchta, 2005), the ability to use this mechanism for targeting greatly expands the range of tissues and explants available for genome editing. Second, as described above, detection and sequence verification is much more straightforward for NHEJ‐derived targeted events than for HDR‐derived targeted events, particularly in the case of increasingly standard NGS‐based analytical methods. Third, the ability to target tissues possessing only NHEJ activity may allow the use of donors with repeated elements, which might otherwise undergo rearrangments were they only able to be targeted in tissues possessing potent HDR activity. Finally, and perhaps most important to implementation, it is possible that NHEJ‐based targeting may be capable of inserting much larger donors than HDR‐based targeting, as donors are simply ligated directly into a DSB, and do not require replication by a processive polymerase.

Consistent with the possibility that NHEJ can be effectively used to target large fragments of exogenous DNA, we have demonstrated here the targeted integration of a donor that both lacks long homology arms and is sufficiently large, at 16.2 kb, to carry four functional transgenes. The need to deliver donors in this size range has become more acute as researchers in academia and industry aim to engineer loci encoding complex, multigene traits or stacks of simple traits, such as those conferring herbicide tolerance or insect resistance (Petolino and Kumar, 2016). While donors as large as 21 kb have been targeted in mammalian cells (Jiang *et al*., 2013), the size of donors targeted in plants has generally been less than needed for this type of locus engineering (Petolino and Kumar, 2016). The apparently lower frequency with which we obtained intact targeted events using the 16.2 kb donor compared to the 7.1 kb donor is possibly attributable to increased donor fragmentation with the larger molecule, consistent with the observation that the larger donor yielded more events with a single junction amplicon, as well as previous suggestions that larger donors fragment more easily (Ercolano *et al*., 2004). Given that we obtained multiple plants with a 16.2 kb donor, it seems likely that even larger donors could be delivered using this method. Alternatively, additional DNA could be added to the same locus with a further round of targeting, for instance to a landing pad like that included in our designs. Which of these approaches would be less time‐ and resource‐intensive is likely a function of the capabilities of the individual laboratory in which the work is performed and the requirements of the project.

Most of the targeted integrations we observed were oriented such that the direction of transcription of the transgenes was in the same orientation as the transcription of FAD2‐1a. This effect may result from annealing of the complementary *Fok*I‐derived overhangs in the genome and the target left by the action of the ZFN, consistent with previous work showing increased targeting frequency of a linear donor with complementary overhangs to a ZFN‐induced DSB (Orlando *et al*., 2010). If this is the case, then NHEJ‐mediated, orientation‐selective targeting should also be attainable in plants using other available engineered nucleases, such as TALENs (which typically use *Fok*I), nuclease‐dead Cas9 pairs fused to *Fok*I (Guilinger *et al*., 2014), or pairs of Cas9 nickases (Shen *et al*., 2014). Alternatively, it may be that in the case of the particular donor and target reported here, integration in this orientation is more favourable for the recovery of transformants, for example, by supporting more robust or stable expression of HPT. The fact that the majority of forward orientation events possessed intact or nearly intact reconstitutued ZFN‐binding sites (Fig. 3d) is consistent with a model in which ZFN activity and donor DNA exist in the same cell only transiently, though we cannot at present assess the number of targeting events that may have occurred but gone unobserved due to subsequent re‐excision.

This report is one of only a few demonstrating gene targeting in soybean, and represents a significant advancement from previous results. Li *et al*. (2009) previously reported the targeted integration of a biolistically delivered, two‐transgene donor to a target site of embryogenic tissue culture cells using FLP recombinase. Critically, this approach, unlike that we report here, required pre‐integration of a transgenic cassette containing a FLP target site. An additional difference is that the FLP‐based approach utilized a donor with a promoterless selectable marker to specifically select for targeting. While this donor design clearly facilitates the identification of targeted events, it is unlikely to be an option in the case of targeting to an endogenous locus. More recently, Li *et al*. (2015) reported HDR‐mediated, targeted integration of a biolistically delivered donor to two different target endogenous soybean loci using CRISPR/Cas. Although this approach did not involve selection for targeting, only a short, single‐transgene donor was delivered to the target locus, in contrast with the considerably larger, four‐gene donor reported here. Furthermore, although the frequency of targeted integration in primary calli reported in the CRISPR‐based approach was encouraging, many of the subsequently regenerated T_0_ plants were either chimeric or had lost the targeted integration completely, complicating the task of obtaining stable, heritable targeted events. In contrast, we did not observe any evidence of chimerism in the events we have reported here, nor did this appear to be a significant issue in the earlier FLP‐based experiments, suggesting that this issue may arise as a result of differences in the methods of transgene delivery or regeneration of mature plants.

Of critical importance to any future application of gene targeting technology in plants is the ability to generate mature, fertile plants with the desired genome modifications, at a cost not prohibitive in time and resources. From the work reported here, we have obtained several mature, targeted plants for each of three configurations of donor and ZFN expression constructs (7.1 kb donor, homodimerizing *Fok*I; 7.1 kb donor, heterodimerizing *Fok*I; and 16.2 kb donor, heterodimerizing *Fok*I), with a required input of 5 000 to 10 000 bombarded embryos in each case. This number of bombardments, though substantial, is well within the number that might occur for a commercial or larger scale research soybean transformation campaign, and would be reduced further by future improvements increasing transformation or regeneration frequency, or reducing donor fragmentation. The purported error‐prone nature of NHEJ did not present a significant problem in this system, as the great majority of genome‐donor junctions we observed were either perfect or near‐perfect.

The system presented here fits within a broader effort to develop and reduce to practice effective genome editing technologies for crop plants, particularly maize and soybeans. Elements of this platform now in place include (i) transformation methods, (ii) a suite of engineered nucleases to generate DSBs at desired loci, (iii) validated donor configurations for either HDR‐ or NHEJ‐mediated integration, (iv) analytical methods for identifying and characterizing targeted events and (v) a bioinformatics‐derived set of potentially attractive targeting sites (Sastry‐Dent *et al*., 2018, 2015). The demonstration of targeted integration of a large, multigene donor to an endogenous soybean locus via NHEJ represents a major step towards the realization of crop improvement via targeted transgene integration. Continuing to build upon and optimize this set of tools for gene targeting, while further reducing resource requirements remains fertile ground for future investigation.

## Experimental procedures

### Vectors

The characterization of the ZFN monomer pair used in this study, and its selection from several candidate ZFN monomer pairs targeting *FAD2‐1a*, have been described previously (see information for monomers 37370 and 37371 in Ainley *et al*., 2016). Vectors expressing homodimerizing and heterodimerizing *FAD2‐1a*‐specific ZFNs were constructed from the previously reported vectors pDAB115601 and pDAB115605 (Ainley *et al*., 2016), respectively, by replacing the Cassava vein mosaic virus (CsVMV) promoter with that of Agrobacterium *MANNOPINE SYNTHASE* (Langridge *et al*., 1989; Velten *et al*., 1984). The two ZFN monomers are generated from the single transcript by virtue of the intervening 2A sequence from *Thosea asigna* (Fang *et al*., 2005; Miller *et al*., 2007). Each monomer possesses an amino‐terminal nuclear localization signal from maize *opaque‐2* (Maddaloni *et al*., 1989). The HDR donor vector contains the HPT coding sequence under the control of the 5′ and 3′ transcriptional regulatory elements from Arabidopsis *UBI10* (AtUBI10; At4g05320) and *Agrobacterium tumefaciens ORF23* (AtuORF23)(Barker *et al*., 1983), respectively, flanked distally by 1001 bp homology arms from *FAD2‐1a*. The 7.1 kb NHEJ donor vector contains HPT under the control of CsVMV and AtuORF23 elements and TurboRFP (Evrogen, Moscow, Russia) under the control of AtUBI10 and AtuORF23 elements. The 16.2 kb NHEJ vector contains HPT under the control of CsVMV (Verdaguer *et al*., 1998) and the 3′ regulatory element of Agrobacterium *ORF1* (Barker *et al*., 1983), a glyphosate tolerance marker (dgt‐28) under the control of Arabidopsis *UBQ14* (At4g02890) regulatory elements, and glufosinate and 2,4‐D tolerance markers (dsm‐2 and aad‐1 respectively) under the control of AtUBI10 regulatory elements.

### Biolistic transformation of embryogenic tissue culture

Soybean embryogenic tissue cultures from two cultivars, Westag 97 and X5 (Agriculture and Agri‐Food Canada breeding line X2650‐7‐2‐3) were initiated, maintained and bombarded as previously described (Simmonds, 2003), with each bombardment involving five or six ~150 mg embryogenic clusters and 0.5 mg of gold particles carrying 8 nmol of donor construct and either 1.6 or 3.2 nmol of ZFN expression construct, as indicated by the molar ratios reported in the results. Bombarded tissues were allowed to recover in liquid medium lacking hygromycin for ~2 weeks, then subjected to ~2 weeks of hygromycin selection at 30 mg/L, and maintained in hygromycin at 55 mg/L thereafter. Green colonies, representing individual events, were identified and isolated over the subsequent 4‐8 weeks.

### Direct transformation of immature soybean embryos

Direct transformation of immature soybean embryos was performed essentially as described previously (Chennareddy *et al*., 2018). Briefly, pods of soybean cv. Maverick (Sleper *et al*., 1998) were collected from greenhouse‐grown plants 7‐14 days after flowering. Pods were incubated for 7 days at 4 °C, surface sterilized with 10% bleach, and dissected to isolate seeds. Seeds were bisected along the hilum and seed coats were removed, yielding two split embryo explants. Split embryo explants were placed onto solid plasmolysis medium (4.4 g/L Murashige & Skoog [Murashige and Skoog, 1962] Basal Medium with Vitamins [Product M519, PhytoTechnology Laboratories, Shawnee Mission, KS, USA], 2.3 g/L Gelrite, 0.4 m mannitol, 0.4 m sorbitol, 0.5 mm MgCl_2_) and maintained in a Conviron growth chamber (24 °C, 18/6 h light/dark photoperiod) for all remaining culture steps. After 4 h on plasmolysis medium, explants were subjected to biolistic bombardment. After 1 d recovery, explants were cultured 1 week on SE40 medium (4.3 g/L Murashige & Skoog Basal Medium, 1x Gamborg B5 vitamins [Gamborg *et al*., 1968], 30 g/L sucrose, 2 g/L Gelrite, 40 mg/L dichlorophenoxyacetic acid [2,4‐D]) to induce somatic embryogenesis, then 3 weeks on SE40 medium + 10 mg/L hygromycin to select for transformants.

After selection and induction on SE40 medium, the explants were placed on somatic embyrogenesis germination medium (SEGM; equivalent to SE40 minus 2,4‐D) for 6 weeks (subcultured every 2 weeks). By the end of the 6 weeks, the somatic embryos were isolated from the immature embryos from which they had developed and were allowed to mature for another 2 weeks on SEGM. Matured somatic embryos were dehydrated for 5 days in a 15 × 100 mm Petri dish with a 1 cm^3^ piece of SEGM. Dehydrated somatic embryos were then incubated on SEGM for 4–8 weeks (subcultured every 2 weeks) until roots grew and apical meristem had formed. After the shoots had elongated to about 7.5 cm and had a sufficient root system, they were transferred to soil and grown for 2–3 weeks in a Conviron growth chamber. Surviving plants were transferred to large pots and self‐fertilized to produce T_1_ progeny.

### Copy number determination

qPCR analyses were performed with PerfeCTa qPCR Supermix (Quanta Biosciences, Beverly, MA, USA) on a LightCycler 480^®^ Real‐Time PCR System (Roche Diagnostics, Indianapolis, IN, USA) according to the manufacturer's instructions. About 10 ng of genomic DNA was used as template for each reaction, with the following programme: 95 °C 10 min; 35 cycles of 95 °C 10 s, 60 °C 1 min, plate read. Previously identified hemi‐ or homozygous plants were used as standards for each transgene.

### Junction PCR and amplicon sequencing

PCR reactions were set up using Takara ExTaq DNA polymerase (Takara Bio, Otsu‐shi, Shiga, Japan) according to the manufacturer's instructions. Approximately 4 ng of genomic DNA was used as template for each reaction, with the following thermalcycler programme: 95 °C for 3 min; 35 cycles of 95 °C 30 s, 60 °C 30 s, 72 °C 4 min. Sequencing was provided by Eurofins Genomics (Louisville, KY, USA). PCR and sequencing primers were designed using the online Primer3 tool (v. 4.1.0) (http://bioinfo.ut.ee/primer3). Amplicon sequences were aligned and analysed with the software tool Sequencher™ (v. 4.9; Gene Codes Corporation, Ann Arbor, MI).

### Sequence capture and Illumina sequencing

Illumina library production from sonicated genomic DNA, size‐selected to ~800 bp was performed as described (Guttikonda *et al*., 2016). Libraries were subjected to hybridization‐based sequence capture using a custom sequence capture kit (Roche NimbleGen, Madison, WI) according to the manufacturer's instructions. The 150 bp paired‐end (PE) reads were obtained using an Illumina MiSeq sequencer in 48‐plex pools. Read trimming, filtering and mapping were performed as described (Guttikonda *et al*., 2016).

## Supporting information


**Table S1** Summary of stringent junction PCR and NGS on 62 candidate targeted events from NHEJ‐based targeting experiments with embryogenic suspension cells.
