# Supplementary material for: Zinc finger nuclease‐mediated targeting of multiple transgenes to an endogenous soybean genomic locus via non‐homologous end joining
Source: Plant Biotechnol J. 2018 Oct 15;17(4):750–61. doi: 10.1111/pbi.13012 (PMC6419576; doi:10.1111/pbi.13012)
Supplement: Supplementary file 1 — Table S1 Summary of stringent junction PCR and NGS on 62 candidate targeted events from NHEJ‐based targeting experiments with embryogenic suspension cells. [file PBI-17-750-s001.docx]

Supplemental table I. Summary of stringent junction PCR and NGS on 62 candidate targeted events from NHEJ-based targeting experiments with embryogenic suspension cells. For each event (NHEJ-02 through NHEJ-63), a + indicates the presence of an amplicon of a size consistent with an intact donor, derived from either the 5′ or the 3′ genome-donor junction, in the orientation indicated. A gray shaded box indicates the detection of an NGS read indicative of a targeted insertion at FAD2-1a at the junction and orientation indicated.
